# Extraspinal ossifications after implantation of vertical expandable prosthetic titanium ribs (VEPTRs)

**DOI:** 10.1007/s11832-014-0585-0

**Published:** 2014-04-22

**Authors:** Vanja Zivkovic, Philippe Büchler, Dror Ovadia, Rolf Riise, Ralf Stuecker, Carol Hasler

**Affiliations:** 1 Gasstr. 65, 4056 Basel, Switzerland; 2 Institute for Surgical Technology and Biomechanics, University of Bern, Stauffacherstrasse 78, 3014 Bern, Switzerland; 3 Department of Paediatric Orthopaedics, Tel Aviv Sourasky Medical Center, Dana Children’s Hospital, Tel Aviv, Israel; 4 Orthopaedic Department, Oslo University Hospital, Nydalen, Postboks 4950, 0424 Oslo, Norway; 5 Altona Children’s Hospital, Hamburg, Germany; 6 Orthopaedic Department, University Children’s Hospital, Spitalstrasse 33, 4031 Basel, Switzerland

**Keywords:** VEPTR, Ossifications, Retrospective, Radiographs, Multicentre

## Abstract

**Introduction:**

Though developed for thoracic insufficiency syndrome, the spinal growth-stimulating potential and the ease of placement of vertical expandable titanium ribs (VEPTRs) has resulted in their widespread use for early-onset spine deformity. Observation of implant-related ossifications warrants further assessment, since they may be detrimental to the function-preserving non-fusion strategy.

**Patients and methods:**

Radiographs (obtained pre and post index procedure, and at 4-year follow-up) and the records of 65 VEPTR patients from four paediatric spine centres were analysed. Ossifications were classified as type I (at anchor points), type II (along the central part) or type III (re-ossification after thoracostomy).

**Results:**

The average age at the index procedure was 6.5 years (min 1, max 13.7). The most prevalent spine problem was congenital scoliosis (37) with rib fusions (34), followed by neuromuscular and syndromic deformities (13 and 8, respectively). Idiopathic and secondary scoliosis (e.g. after thoracotomy) were less frequent (3 and 4, respectively). Forty-two of the 65 (65 %) patients showed ossifications, half of which were around the anchors. Forty-five percent (15/33) without pre-existing rib fusions developed a type II ossification along the implant. Re-ossifications of thoracostomies were less frequent (5/34, 15 %). The occurrence of ossifications was not associated with patient-specific factors.

**Conclusions:**

Implant-related ossifications around VEPTR are common. In contrast to harmless bone formation around anchors, ossifications around the telescopic part and the rod section are troublesome in view of their possible negative impact on chest cage compliance and spinal mobility. This potential side effect needs to be considered during implant selection, particularly in patients with originally normal thoracic and spinal anatomy.

## Introduction

The goals of any growth-promoting operative strategy for the treatment of early-onset spinal deformity (EOS) are the beneficial alteration of the natural history of associated cardiopulmonary deficiencies and the underlying spinal deformity as well as the prevention of a negative change in the spinal biomechanics due to the immobilizing effect of the implant [3, 22]. The latter aim is reflected in the term “non-fusion”. This descriptor is assigned to any growth-sparing procedure in which there is believed to be an absence of autofusion, bridging ossifications and negative effects on the facet joints and the discs.

Vertical expandable prosthetic titanium ribs (VEPTRs) are mostly extraspinal implants that qualify as non-fusion procedures. Traditionally, the primary reasons for their application have been to treat thoracic insufficiency syndrome and improve survival rates by chest expansion [5–8]. However, promotion of spinal growth and deformity correction, even for severely jumbled spines, are welcome side effects [4]. Although still controversial among spine surgeons, there is an emerging consensus that spine-based strategies are preferable for normally segmented spines, while VEPTR is the treatment of choice for congenital malformed spines and thoraces [33, 34]. Nevertheless, VEPTR has also gained popularity as a treatment for a range of non-congenital EOSs without concomitant thoracic pathologies. Its extraspinal placement is thought to result in less neurologic risk, less damage to the facet joints and paraspinal musculature, and therefore preservation of the biomechanical function of the spine [16, 27, 30, 37]. In contrast to distractible spine-based constructs, such as growing rods or passive growth-guiding constructs (Luque or Shilla type), VEPTR is also believed to overcome spinal autofusion [2, 19].

In many spine centres, VEPTR has been used for 5–10 years. Accordingly, many of the early patients have undergone more than 10 expansion procedures, changes of implants and also some unplanned surgeries, mainly for cradle dislodgements, skin sloughs and infection [3, 13, 16, 26, 31, 36]. Given that there are an increasing number of VEPTR patients who are reaching skeletal maturity with a relative stiff spine at the time of conversion into instrumented fusion, underlying autofusion and ossifications must be hypothesized [12, 18]. It has been our anecdotal experience that unwanted new bone formation adjacent to the implant makes the term “non-fusion” and the assumption of function preservation questionable. However, there is a paucity of literature on those topics. Groenefeld and Hell recently claimed that the radiographic occurrence of ossification rose continuously up to 48 % at 53 months after the index procedure in a single-centre study of 57 VEPTR patients [14].

In an attempt to shed further light on those findings, we studied the type and occurrence of heterotopic and periprosthetic ossifications 4 years after VEPTR implantation in a cohort of patients recruited from four international VEPTR centres.

## Materials and methods

### Inclusion criteria

After approval by the institutional review boards at the four spine centres involved, we conducted a retrospective radiologic study on a subset of patients who underwent a VEPTR implantation at least 4 years previously, irrespective of the underlying diagnosis. The decision to perform a cross-sectional study at this time point was made based on independent case-sensitive observations on the occurrence of ossifications at each centre, intraoperative force measurements taken when growing rods were expanded, which indicated a linear increase in stiffness during the first 3–4 years of expansion, and the recently reported radiographic incidence of ossifications over time [14, 24]. Inclusion criteria comprised complete radiologic documentation, including standard anteroposterior and lateral views after the index procedure and 4 years thereafter. Patients with a history of spinal surgery prior to the VEPTR implantation were excluded. Serial device expansions at scheduled intervals of 6 months were commonly performed at all participating centres. Patients had therefore undergone an average of 7–8 such procedures and at least one replacement of the implant.

### Data acquisition

Each centre provided anonymized patient data and digitized spine radiographs, which were reviewed on a PACS workstation by a VEPTR-experienced spine surgeon from one of the study centres (CH) and a medical student (VZ). Patient-specific preoperative variables such as gender, age at the time of VEPTR implantation, underlying diagnosis, osteotomy of fused ribs (thoracostomy) and type of VEPTR construct were retrieved for all patients. We did not include curve severity nor the occurrence of infection since Cobb angle measurement is inaccurate in cases of severe congenital deformity, and clinically nonapparent implant colonization may have also contributed to bone formation but was not analyzed [15].

### Radiographic analysis

The first erect radiograph after VEPTR implantation served as an ossification-free baseline. The primary outcome parameter was the presence or absence of an ossification on the standard radiographs at the 4-year follow-up, independent of the size. For some patients there were CT scans that clearly showed an ossification despite a normal radiographic appearance. Those patients were rated as having no ossification, since we conducted a radiographic study. Only a minority of the patients had CT scans, and performing CT scans of all patients to detect ossifications was deemed unethical due to the radiation exposure.

**Fig. 1:**
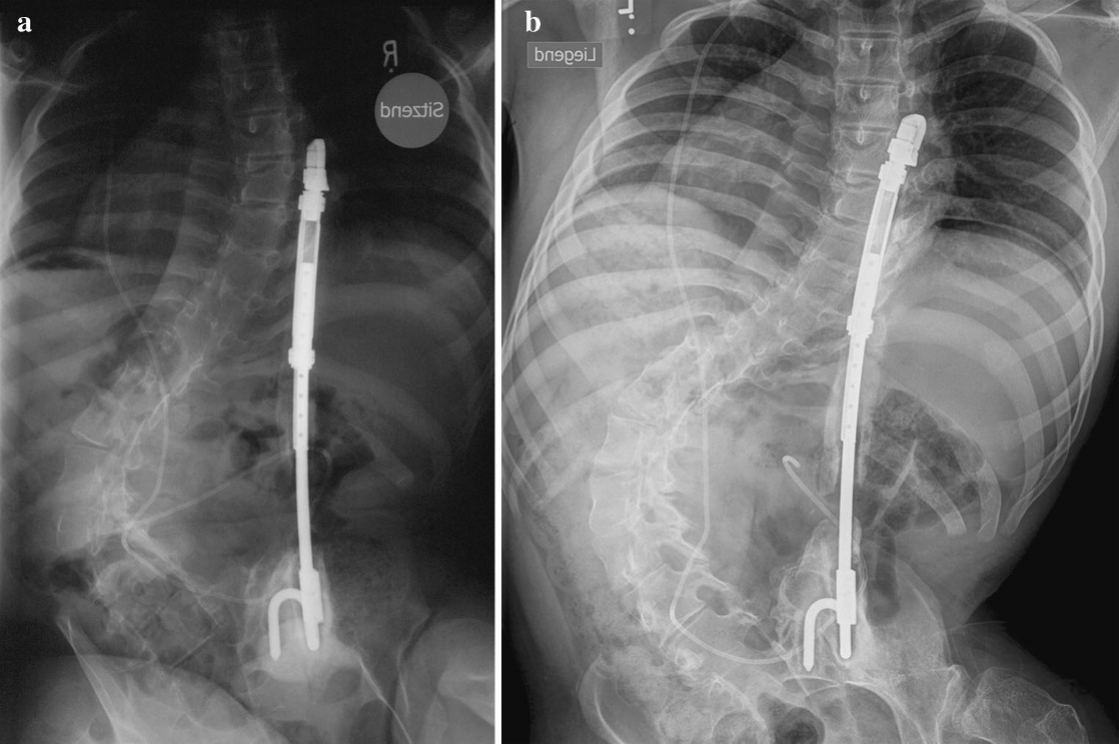
**a** Sixteen-year-old wheelchair-bound boy with neuromuscular scoliosis (myelomeningocele) 4 years after unilateral VEPTR implantation: new bone formation at the ileum anchor point (type Ic) and along the titanium rib at the thoracic level (IIa) and the lumbar rod section (IIb). **b** Seven years after the index procedure there is an almost continuous bone mass along the implant, reaching from the rib cradle to the ala hook

**Fig. 2:**
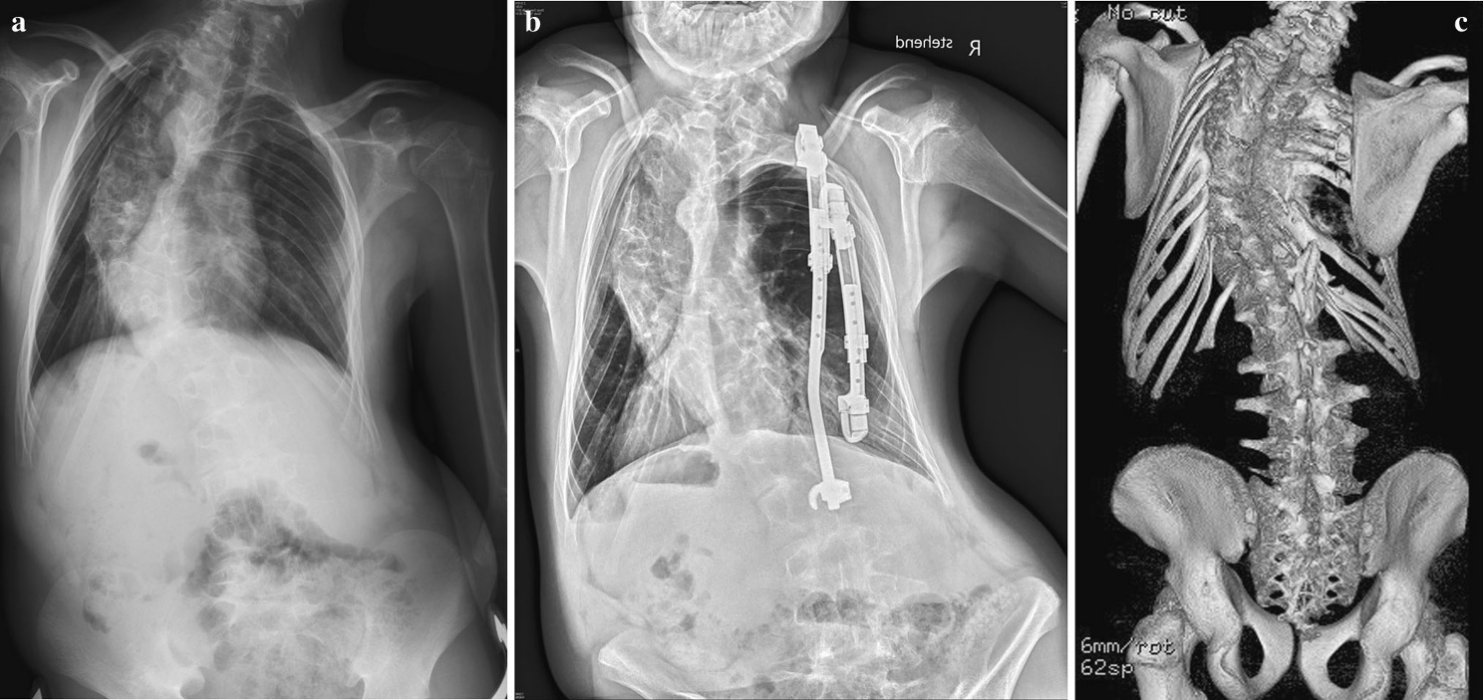
**a** Twelve-year-old boy with congenital scoliosis prior to the index procedure. **b** Four years later, new bone formation along the rod section of the inner VEPTR constructs was suspected. **c** A CT 7 years later (at the age of 19 years) confirmed the presence of ossifications at the level of the former lamina hook (Ib) reaching over the caudal ribs (IIa), and also at the level of the former upper rib cradle (Ia). The thoracostomy remained open even after removal of the VEPTR. Based on those findings, a decision was made not to instrument and fuse the spine, since the situation was deemed stable

The ossifications on the follow-up radiographs were categorized as follows (Figs. 1, 2):

Type I: at anchor points (A. rib cradle, B. lamina hook, C. ala hook)

Type II: along titanium rib (A. over the ribs, B. at the lumbar level)

Type III: re-ossification after rib osteotomy (opening-wedge thoracostomy [6]).

### Statistical analysis

A simulation-based resampling approach was applied to estimate sample size. The effect per patient and curve variable was defined as the increase in the probability of having an ossification 4 years after VEPTR implantation. For this study, 288 patients had to be recruited to achieve a power of 1 − *β* = 0.9 at a significance level of *α* = 0.05.

The statistical calculations were performed with the open-source package R (http://www.r-project.org/). Fisher’s exact test was used to determine if the proportion of ossification was the same in males and females. Similarly, pairwise Fisher tests were used to compare the centres in terms of proportion of ossification and to compare the different types of spinal deformity. To correct for multiple significance tests, the Holm method was used to adjust the *p* values and preserve family-wise type I (or false-positive) errors. Two-sided *t* tests were used to compare the age distributions of patients with and without ossification. Statistical significance was defined as *p* < 0.05.

## Results

The average age of the 65 patients at the time of the index procedure was 6.5 years (min 1, max 13.7 years), and there was an almost balanced female:male ratio (31:34). The most prevalent underlying spine problem was a congenital scoliosis with multilevel malformations (37) and hemithorax constriction due to rib fusions (34/37), followed by neuromuscular and syndromic deformities (13 and 8, respectively). Idiopathic and secondary early-onset scoliosis (e.g. after thoracotomy) was less frequent (3 and 4, respectively). All patients underwent a routine half-yearly expansion program, resulting in 7–8 lengthening procedures during the 4-year observation period.

No heterotopic ossifications were detected (these are bone formations which are not in contact with any part of the implant, and do not occur in the regions of former osteotomies of congenital rib fusions).

The incidence of each type (I–III) of ossification is given in Table 1. Due to less overlap with anatomical structures and the contralateral implant, half of the bone formations (22/42) were only detectable on the anteroposterior projection, a few (3/42) were seen only on the lateral projection, and the remainder were noted in both projections (17/42). In total, 42 of 65 (65 %) patients showed at least one ossification. Half of the 119 ossifications occurred around the anchors (Table 1). Almost half of the patients (15/33) without pre-existing congenital rib fusions developed an ossification along at least one of the rib sleeves of their rib-to-rib, rib-to-spine or rib-to-pelvic implants. The occurrence of ossifications was not statistically associated with patient-specific factors such as age, gender, underlying disease and the nature of the spinal deformity.

**Table 1:**
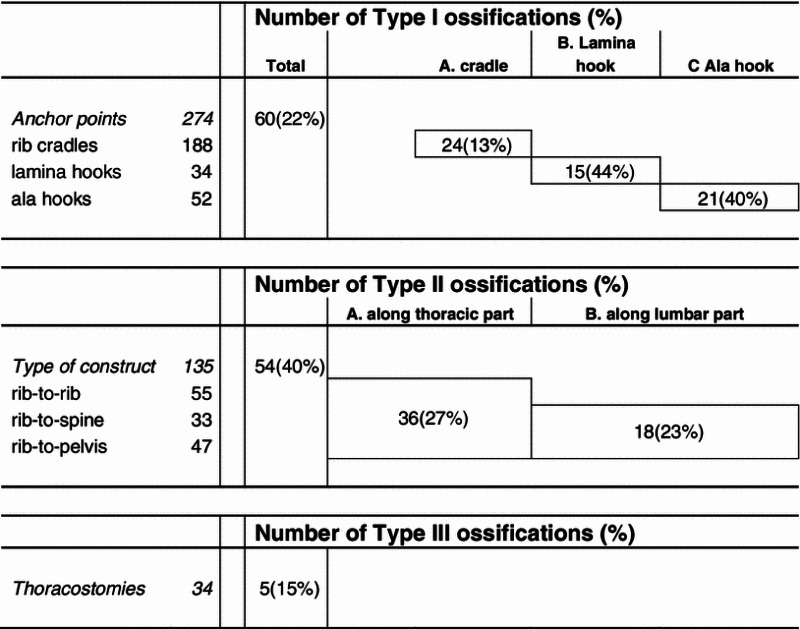
Type and incidence of ossifications in 65 VEPTR patients

## Discussion

An extraspinal, implant-related bone mass contradicts the concept of a fusionless treatment strategy.

Ossifications around implants in non-fusion constructs for early-onset spine deformities occur but are underreported [14, 21]. Two-thirds of our patients showed ossifications, which is a slightly higher rate than previously reported [14]. Half of those ossifications were situated around the anchors. Half of the patients without pre-existing rib fusions developed ossifications along the implant overlying the ribs. Re-ossifications of thoracostomies were less frequent (15 %). Undue pooling of those subtypes results in a high incidence of a generally harmless issue, so there is a need to categorize, as pathogenesis and functional and therapeutic sequelae may vary.

### Type I ossifications

A slow asymptomatic drift of a cradle through the rib or a local fracture of lamina or ala, disengagement and skin sloughs are well-known reasons for unplanned surgery, but represent solvable problems with little or no long-term impact [8, 16, 17, 36]. We detected local bone formations around VEPTR anchor sites at ribs, the lumbar spine and the ala in every fifth patient. We consider them to be mainly a harmless biologic reaction to a creeping cutout or to the polyaxiality of the implant anchor and to the intended unrestricted motion of the patient during daily activities and sports within the framework of the non-fusion philosophy. However, in the case of laminar hook migration, ossification around the facet joint may lead to loss of a motion segment and the need to extend the instrumentation caudally at the time of definitive fusion [18]. If the bone is strong enough to withstand the local forces, it is subjected to repeat peak forces at the implant–bone interface. Local bone formation has to be regarded as a means to increase this contact surface and decrease the local force in order to provide more stable seating of the cradles and hooks. Alternatively, local sclerosis is seen when an ala hook sinks deeper into the ileum. Trunk, spine and chest wall biomechanics explain the different incidences of local ossification at the VEPTR fixation points: it is more than three times higher for the caudal anchors (lamina 44 %, ala 40 %) compared to the rib cradles (13 %). This contrasts with a previous report which found that most problems occur at the lumbar spine [14]. The cone of trunk motion, with its caudal basis, entails much more motion at caudal than at cranial anchors. Moreover, the cradles are fixed on relatively mobile ribs, while the seating of lumbar hooks and ala hooks is firmer and therefore exerts higher stress on a bigger bony surface [10]. On the other hand, rib cradles have a smaller contact zone and tend to cut through. The stiffness of the curve seems to play an important role, since less flexible spines require higher distraction forces, which cause more implant migration [14].

### Type II ossifications

Bone formations along the main central part of the implant (extension bar, lumbar extension rod) occurred in more than half of the patients. The causes remain hypothetical: patient characteristics such as the individual potential for bone formation, extensive dissection, local haematoma along the implant within the subfacial-submuscular tunnel, damage to the soft tissues or rib periosteum, bacterial colonization and infection, recurrent surgery and local inflammation, as well as the implant’s bulkiness and material properties may all play a role [14, 20, 29, 32, 35]. Type II ossifications are the most troublesome, since they may lead to either direct stiffening of the thorax or restricted spine motion (although they are remote from the spine), which may limit the effect of further device expansion over time by acting as a powerful lateral tether [29]. This phenomenon, called “the law of diminishing returns”, is also encountered in patients with spine-based expandable implants [24, 29].

Ossifications of the thoracic wall deserve special attention. Half of the patients without pre-existing congenital rib fusions developed an ossification along the extension bar of their rib-to-rib, rib-to-spine or rib-to pelvic implant. This may have little or no immediate effect on the compliance of the rib cage as long as the stiff titanium implant is in situ, but an intercostal bony bridge hinders lengthening and may affect regional chest wall motion and global chest wall compliance. It should therefore be removed at the time of implant expansion [9]. Instead of an easy, short expansion operation over a small skin incision, exposure and—occasionally—temporary removal of the implant might be indicated in order to provide full access to the ossification and its complete removal. With new-generation magnetic implants, this would even mean an unwanted return to the operating room. There are currently no data on the re-occurrence of such a bridge, the potential effect of remaining fibrous scars and the benefit of removal on pulmonary function. In cases with pre-existing congenital rib fusions, the effect on chest wall compliance might not be as pronounced as in cases with previously normal anatomy. However, it corrupts the goal of maximal thorax expansion during the course of repeat VEPTR lengthening over the years. Type II ossification may also play an important role when it comes to removal of the VEPTR towards the end of growth with or without instrumented fusion of the affected levels. They may not be recognized and—apart from autofusion due to the immobilizing effect of any spinal and paraspinal implant—may compromise the final correction of the deformity [1, 18]. When the affected levels are not included in the definitive fusion at the end of spinal growth, motion will be restricted.

### Type III ossifications

Only 15 % of the patients who had undergone an opening-wedge thoracostomy at the time of the index procedure showed re-closing of the intercostal gap. These ossifications are re-fusions and are not directly related to the implant but rather to the proximity to bleeding bone surfaces and the stability after VEPTR placement. We recommend a careful preoperative look at the radiograph. In cases of suspected re-ossification, a CT scan should be performed. Intraoperatively, the implant should be checked for adjacent ossifications. If confirmed, a re-osteotomy at the time of scheduled implant lengthening provides optimal expansion of the thorax [11].

### Strengths and limitations of this study

This study has demonstrated that peri-implant ossifications are common in VEPTR patients. However, the multicentre and retrospective nature of the study leads to limitations such as data quality control, missing or inadequately collected data and variations in radiographic quality. We could not correlate the occurrence of unwanted bone formation with predictive patient characteristics, mostly due to a lack of statistical power. According to our sample size analysis, a study population of more than 280 VEPTR patients would have been necessary. Since less than half of the VEPTR patients in each centre reach the required 4 years of follow-up, this would mean the involvement of additional centres or a long-term prospective study. Both of these approaches are not feasible options for obtaining valid data within a useful timeframe, though we expect a further rise in the incidence of ossification over time. It is often difficult to determine whether new ossifications adjacent to the implant or re-fusion of thoracostomy have/has occurred. Variable radiograph quality is a limiting factor, with frequent overexposure occurring in two centres. This leads to potential underrecording of ossifications. In addition, radiographs are a less sensitive modality for detecting bone formation than CT scans. However, due to the extraspinal position of VEPTRs, meaning that there is no overlap with the spine, ossifications are still easier to detect with VEPTRs than with growing rods in place [21]. The reported incidences are therefore much more likely to be too low. In addition, local scarring is not detectable with imaging. We firmly believe that type II ossifications negatively impact chest wall compliance. However, no objective data are available to confirm this assumption.

## Conclusions

Implant-related ossifications after VEPTR operations for early-onset spine deformities (EOS) are common and occur within the first years after the index procedure [14]. Our findings aid understanding of the possible limitations of either extra- or juxtaspinal growth-retaining stiff implants [1, 16, 19, 24, 25, 28, 29]. Stabilizing bone formation around the rib-, spine- and ileum-based anchor points is a seemingly harmless local reaction to the polyaxiality and the non-fusion strategy. In contrast, ossifications around the central telescoping part and the rod section of the construct are troublesome since they bridge ribs and spinal segments, leading to potential negative impacts on chest cage compliance and spinal mobility. This phenomenon of unwanted extraspinal fusion needs to be considered when the implant employed to correct the EOS is chosen, since it may corrupt the non-fusion philosophy, particularly in patients with originally normal thoracic and spinal anatomy. Resection of such ossifications when the central portion is replaced or at the time of conversion into a definitive instrumented fusion should be taken into consideration and weighed against the option of leaving it as a precursor and alternative to the final fusion [21, 24].

There has been increasing interest in the use of fusionless techniques to treat early-onset spine deformities. Whether our results apply to other forms of growing instrumentation such as growing rods remains unknown. Curve stiffness was reported to correlate with the occurrence of ossifications [14]. Stiffness of the implant may as well. Efforts are underway to prevent the reported negative impact of prolonged treatment with commonly used distraction-based stiff implants by applying novel methods; for example, flexible tethering of the spine [22, 23]. However, for the time being, simultaneous deformity control, growth modulation and full preservation of the biomechanical integrity of the spine remains a rather distant goal.

## References

[1] Akbarnia BA, Emans JB (2010). Complications of growth-sparing surgery in early onset scoliosis. Spine (Phila Pa 1976).

[2] Cahill PJ, Marvil S, Cuddihy L (2010). Autofusion in the immature spine treated with growing rods. Spine (Phila Pa 1976).

[3] Campbell RM (2013). VEPTR: past experience and the future of VEPTR principles. Eur Spine J.

[4] Campbell RM, Jr, Hell-Vocke AK (2003). Growth of the thoracic spine in congenital scoliosis after expansion thoracoplasty. J Bone Joint Surg Am.

[5] Campbell RM, Smith MD (2007). Thoracic insufficiency syndrome and exotic scoliosis. J Bone Joint Surg Am.

[6] Campbell RM, Jr, Smith MD, Hell-Vocke AK (2004). Expansion thoracoplasty: the surgical technique of opening-wedge thoracostomy. Surgical technique. J Bone Joint Surg Am.

[7] Campbell RM, Jr, Smith MD, Mayes TC (2003). The characteristics of thoracic insufficiency syndrome associated with fused ribs and congenital scoliosis. J Bone Joint Surg Am.

[8] Campbell RM, Jr, Smith MD, Mayes TC (2004). The effect of opening wedge thoracostomy on thoracic insufficiency syndrome associated with fused ribs and congenital scoliosis. J Bone Joint Surg Am.

[9] Davis JT, Long FR, Adler BH et al (2004) Lateral thoracic expansion for Jeune syndrome: evidence of rib healing and new bone formation. Ann Thorac Surg 77:445–448. doi:10.1016/S0003-4975(03)01340-7 10.1016/S0003-4975(03)01340-7 14759413

[10] Dubousset J (2011). Reflections of an orthopaedic surgeon on patient care and research into the condition of scoliosis. J Pediatr Orthop.

[11] Emans JB, Caubet JF, Ordonez CL (2005). The treatment of spine and chest wall deformities with fused ribs by expansion thoracostomy and insertion of vertical expandable prosthetic titanium rib: growth of thoracic spine and improvement of lung volumes. Spine.

[12] Flynn JM, St. Hilaire T, et al. (2008) Is definitive spinal fusion, or VEPTR removal, needed after VEPTR expansions are over? An analysis of the 39 “VEPTR” graduates. In: 43rd Annual Meeting of the Scoliosis Research Society, Salt Lake City, UT, USA, 10–13 Sept 2008

[13] Giehl J (2008) VEPTR—effectivity and safety (abstract). Eur Spine J 17(11):1622

[14] Groenefeld B, Hell AK (2013). Ossifications after vertical expandable prosthetic titanium rib treatment in children with thoracic insufficiency syndrome and scoliosis. Spine (Phila Pa 1976).

[15] Hasler CSD, Trampuz D, Plaass C (2013). Bacterial colonization of spine implants in children with severe spinal and thoracic deformities treated with growth retaining implants. J Child Orthop.

[16] Hasler CC, Mehrkens A, Hefti F (2010). Efficacy and safety of VEPTR instrumentation for progressive spine deformities in young children without rib fusions. Eur Spine J.

[17] Hell AK, Campbell RM, Hefti F (2005). The vertical expandable prosthetic titanium rib implant for the treatment of thoracic insufficiency syndrome associated with congenital and neuromuscular scoliosis in young children. J Pediatr Orthop B.

[18] Lattig F, Taurman R, Hell AK (2012) Treatment of early onset spinal deformity with VEPTR: a challenge for the final correction spondylodesis: a case series. J Spinal Disord Tech (Epub ahead of print) 10.1097/BSD.0b013e31826eaf27 27196004

[19] Mardjetko SM, Hammerberg KW, Lubicky JP (1992). The Luque trolley revisited: review of nine cases requiring revision. Spine.

[20] Martinez MD, Schmid GJ, McKenzie JA (2010). Healing of non-displaced fractures produced by fatigue loading of the mouse ulna. Bone.

[21] McElroy MJ, Sponseller PD, Dattilo JR (2012). Growing rods for the treatment of scoliosis in children with cerebral palsy: a critical assessment. Spine (Phila Pa 1976).

[22] Newton PO, Farnsworth CL, Upasani VV (2011). Effects of intraoperative tensioning of an anterolateral spinal tether on spinal growth modulation in a porcine model. Spine (Phila Pa 1976).

[23] Newton PO, Upasani VV, Farnsworth CL (2008). Spinal growth modulation with use of a tether in an immature porcine model. J Bone Joint Surg Am.

[24] Noordeen HM, Shah SA, Elsebaie HB (2011). In vivo distraction force and length measurements of growing rods: which factors influence the ability to lengthen?. Spine (Phila Pa 1976).

[25] Pratt RK, Webb JK, Burwell RG (1999). Luque trolley and convex epiphysiodesis in the management of infantile and juvenile idiopathic scoliosis. Spine.

[26] Ramirez N, Flynn JM, Serrano JA (2009). The vertical expandable prosthetic titanium rib in the treatment of spinal deformity due to progressive early onset scoliosis. J Pediatr Orthop B.

[27] Reinker K, Simmons JW, Patil V et al (2011) Can VEPTR^®^ control progression of early-onset kyphoscoliosis? A cohort study of VEPTR^®^ patients with severe kyphoscoliosis. Clin Orthop Relat Res 469:1342–1348 10.1007/s11999-010-1697-6 PMC3069267 21116753

[28] Sankar WN, Acevedo DC, Skaggs DL (2010). Comparison of complications among growing spinal implants. Spine (Phila Pa 1976).

[29] Sankar WN, Skaggs DL, Yazici M (2011). Lengthening of dual growing rods and the law of diminishing returns. Spine (Phila Pa 1976).

[30] Smith J, Smith M (2008) Treatment of progressive spinal deformity using the VEPTR device with a bilateral percutaneous rib to pelvis technique in non-ambulatory children with neuromuscular disease (abstract). In: 2nd Int Congr on Early Onset Scoliosis and Growing Spine, Montreal, Canada, 7–8 Nov 2008

[31] Smith JT (2011). Bilateral rib-to-pelvis technique for managing early-onset scoliosis. Clin Orthop Relat Res.

[32] Smith JT, Smith MS (2011). Can infection associated with rib distraction techniques be managed without implant removal?. Spine (Phila Pa 1976).

[33] Thompson GH, Lenke LG, Akbarnia BA (2007). Early onset scoliosis: future directions. J Bone Joint Surg Am.

[34] Vitale MG, Gomez JA, Matsumoto H (2011). Variability of expert opinion in treatment of early-onset scoliosis. Clin Orthop Relat Res.

[35] Waldhausen JH, Redding GJ, Song KM (2007). Vertical expandable prosthetic titanium rib for thoracic insufficiency syndrome: a new method to treat an old problem. J Pediatr Surg.

[36] White KK, Song KM, Frost N (2011). VEPTR growing rods for early-onset neuromuscular scoliosis: feasible and effective. Clin Orthop Relat Res.

[37] Wimmer C, Wallnöfer P (2007) VEPTR—a two year follow up in the treatment of severe spinal deformities (abstract). In: Eur Spine J 16(11):2012

